# Integrating genomics, phenomics, and deep learning improves the predictive ability for Fusarium head blight–related traits in winter wheat

**DOI:** 10.1002/tpg2.20470

**Published:** 2024-06-09

**Authors:** Subash Thapa, Harsimardeep S. Gill, Jyotirmoy Halder, Anshul Rana, Shaukat Ali, Maitiniyazi Maimaitijiang, Upinder Gill, Amy Bernardo, Paul St. Amand, Guihua Bai, Sunish K. Sehgal

**Affiliations:** ^1^ Department of Agronomy, Horticulture and Plant Science South Dakota State University Brookings South Dakota USA; ^2^ Department of Geography & Geospatial Sciences, Geospatial Sciences Center of Excellence South Dakota State University Brookings South Dakota USA; ^3^ Department of Plant Pathology North Dakota State University Fargo North Dakota USA; ^4^ USDA‐ARS, Hard Winter Wheat Genetics Research Unit Manhattan Kansas USA

## Abstract

Fusarium head blight (FHB) remains one of the most destructive diseases of wheat (*Triticum aestivum* L.), causing considerable losses in yield and end‐use quality. Phenotyping of FHB resistance traits, Fusarium‐damaged kernels (FDK), and deoxynivalenol (DON), is either prone to human biases or resource expensive, hindering the progress in breeding for FHB‐resistant cultivars. Though genomic selection (GS) can be an effective way to select these traits, inaccurate phenotyping remains a hurdle in exploiting this approach. Here, we used an artificial intelligence (AI)‐based precise FDK estimation that exhibits high heritability and correlation with DON. Further, GS using AI‐based FDK (FDK_QVIS/FDK_QNIR) showed a two‐fold increase in predictive ability (PA) compared to GS for traditionally estimated FDK (FDK_V). Next, the AI‐based FDK was evaluated along with other traits in multi‐trait (MT) GS models to predict DON. The inclusion of FDK_QNIR and FDK_QVIS with days to heading as covariates improved the PA for DON by 58% over the baseline single‐trait GS model. We next used hyperspectral imaging of FHB‐infected wheat kernels as a novel avenue to improve the MT GS for DON. The PA for DON using selected wavebands derived from hyperspectral imaging in MT GS models surpassed the single‐trait GS model by around 40%. Finally, we evaluated phenomic prediction for DON by integrating hyperspectral imaging with deep learning to directly predict DON in FHB‐infected wheat kernels and observed an accuracy (*R* ^2^ = 0.45) comparable to best‐performing MT GS models. This study demonstrates the potential application of AI and vision‐based platforms to improve PA for FHB‐related traits using genomic and phenomic selection.

## Core Ideas

- Vision and artificial intelligence (AI)‐based technology provide an effective way to phenotype Fusarium‐damaged kernels (FDK) in wheat.
- Inclusion of AI‐based FDK as a covariate in multi‐trait genomic prediction models yields high predictive ability for deoxynivalenol (DON).
- Hyperspectral imaging can be leveraged to improve the predictive ability of DON using genomic prediction as well as for direct phenomic prediction.

**Abbreviations**
AI: artificial intelligence
BLUE: best linear unbiased estimate
CNN: convolutional neural network
CV: cross‐validation
DIS: disease index
DL: deep learning
DON: deoxynivalenol
FDK: Fusarium‐damaged kernels
FHB: Fusarium head blight
GBS: genotyping‐by‐sequencing
GP: genomic prediction
GS: genomic selection
HD: days to heading
HWW: hard winter wheat
MAS: marker‐assistant selection
ML: machine learning
MS: mass spectrometry
MT: multi trait
NIR: near infrared
PA: predictive ability
PCA: principal component analysis
PH: plant height
QTL: quantitative trait loci
SDSU: South Dakota State University
SNP: single‐nucleotide polymorphism
ST: single trait

## 1 INTRODUCTION

Fusarium head blight (FHB), predominantly caused by *Fusarium graminearum* Schwabe, is an economically important fungal disease that adversely affects wheat production in Canada, the United States, and many other countries (Gilbert & Tekauz, 2000; McMullen et al., 2012). FHB‐infected wheat spikes give a premature bleaching appearance and affect kernel development, leading to shriveled kernels with reduced test weight. In addition to grain yield losses, the fungus produces mycotoxins, primarily deoxynivalenol (DON), which pose serious health consequences to humans and livestock if ingested above certain quantities (Miedaner et al., 2017). Different restrictions and limits have been imposed on the maximum level of DON allowed in wheat grains and grain products used for food and feed in most countries including the United States and Canada. In the United States, FHB epidemics have spread to all major wheat‐producing states in the last couple of decades, partially owing to increased maize acreage and reduced tillage (Gilbert & Haber, 2013; McMullen et al., 2012).

Fungicides are frequently used to manage FHB; however, the application of fungicides is quite cumbersome due to a limited application window for the successful management of the disease (McMullen et al., 2012). Further, increased costs and potential environmental impact due to fungicides make the development and deployment of FHB‐resistant cultivars the most effective and environment‐friendly approach to manage and minimize the losses caused by FHB. In the context of FHB, resistance is highly polygenic and influenced by environmental factors. In general, the genetics of FHB resistance have been categorized as type I (resistance to initial infection), type II (resistance to fungal spread across the wheat head), and type III (low mycotoxin accumulation) (Bai et al., 2018). However, only type II resistance has been extensively characterized and exploited in breeding programs as it is the easiest to assess compared with other types (Bai & Shaner, 2004). Furthermore, a large number of quantitative trait loci (QTL) associated with FHB resistance have been identified, including some important genes like *Fhb1*; however, only a few have been successfully incorporated into wheat breeding programs (Bai et al., 2018; Steiner et al., 2017; Venske et al., 2019; J. Zhang et al., 2022b). In the US hard winter wheat (HWW) growing region, native resistance QTL have served as the major source of FHB resistance and played an important role in alleviating HWW FHB losses in the Great Plains (J. Zhang et al., 2022b). Conversely, the small effect of native QTL has limited the applications of marker‐assistant selection (MAS) in developing FHB‐resistant HWW varieties.

In addition to the complex quantitative inheritance, the winter wheat breeders in the Northern Great Plains of the United States face another challenge of shorter turnaround cycles, which makes it almost impossible to evaluate and select for FHB resistance based on postharvest traits, that is, Fusarium damaged kernels (FDK) and DON, which further hinders breeding efforts to develop resistant cultivars (Figure S1). To overcome this limitation, genomic selection (GS) can be an effective alternative that provides an opportunity for the breeders to predict/select for low FDK and DON, which are otherwise challenging to phenotype in an appropriate time frame.

GS is an approach that estimates the genetic worth of an individual based on genome‐wide markers (Heffner et al., 2009; Meuwissen et al., 2001). Rather than relying on a few selected markers as in MAS, GS uses genome‐wide markers in a population jointly to predict the breeding values of individuals (Meuwissen et al., 2001). Plant breeders are increasingly evaluating and adopting GS in their breeding programs (Calvert et al., 2020; Gill et al., 2023, 2021; Lado et al., 2018; Moreno‐Amores et al., 2020). Multiple studies have reported successful evaluation or implementation of GS in different crops, including wheat, indicating the immense potential of GS in plant breeding (Bhat et al., 2016; Gill et al., 2023; Juliana et al., 2017; Lado et al., 2018; Poland et al., 2012). Further, GS has been evaluated and shown promising for FHB resistance in wheat in several studies (Arruda et al., 2015; Dong et al., 2018; Larkin et al., 2020; Mirdita et al., 2015; Rutkoski et al., 2012; Verges et al., 2020; W. Zhang et al., 2021; J. Zhang et al., 2022a). Despite the successful evaluation of GS for FHB resistance in general, the implementation in the breeding programs remains a challenge owing to the lower predictive ability (PA) of genomic prediction (GP) models for these traits.

The PA depends on several factors including the nature and size of training population (TP), accurate phenotyping, trait heritability, and statistical models used (Gill et al., 2021). Accurate and efficient phenotyping for FDK remains a key factor for streamlining the GS in the FHB‐resistance breeding program. However, FDK phenotyping usually uses two traditional approaches involving manual enumeration of FDK by physical separation of healthy and diseased kernels or visual estimation of FDK using a set of standards (Agronomic Crops Network, n.d.). Most breeding programs adhere to the visual estimation as it is relatively faster. However, this method is subjective, highly prone to human bias, and results in inaccurate measurements, lower repeatability, and lower heritability estimates. Subsequently, inaccurate phenotyping remains a bottleneck and limits the exploitation of GS for FDK and DON. Therefore, there is a need to supplement the GP models with emerging phenomics technologies and machine learning (ML) approaches to improve the PA for traits like FDK and DON (Ackerman et al., 2022; Gaire et al., 2022; Rutkoski et al., 2012; Wu et al., 2023).

**Core Ideas**

- Vision and artificial intelligence (AI)‐based technology provide an effective way to phenotype Fusarium‐damaged kernels (FDK) in wheat.
- Inclusion of AI‐based FDK as a covariate in multi‐trait genomic prediction models yields high predictive ability for deoxynivalenol (DON).
- Hyperspectral imaging can be leveraged to improve the predictive ability of DON using genomic prediction as well as for direct phenomic prediction.

In recent years, a few studies have been carried out to evaluate alternative automated and artificial intelligence (AI)‐based methods to obtain FDK employing spectroscopy or image‐based approaches (Ackerman et al., 2022; Barbedo et al., 2015; Delwiche et al., 2019; Maloney et al., 2014; Wu et al., 2023). Notably, Ackerman et al. (2022) used an AI‐powered platform (Vibe QM3i) that uses 2D imaging of kernels in bulk and exploits morphological features to estimate FDK. On the other hand, Wu et al. (2023) incorporated deep learning (DL) to estimate FDK in infected samples. Overall, these studies have shown some improvement in the accuracy of obtaining FDK compared to the manual method, but each approach has its limitations. In the current study, we estimated FDK using a highly accurate AI‐assisted automated sorter (QSorter Explorer) that has been previously used for single kernel–based phenotyping of complex traits in different species (Alvarez et al., 2021; Armstrong et al., 2017; Davis et al., 2021; Rupenyan et al., 2016). QSorter Explorer has been trained to automatically sort the diseased and infected kernels by extracting kernel morphology as well as spectroscopy based on single kernels and enumerating FDK value for the sample using ML models. Here, we assess the PA of GP models for AI‐based FDK in comparison to traditionally estimated visual FDK.

Further, DON is another important trait for improving FHB resistance in wheat. However, quantifying DON levels within grain samples is cumbersome and expensive, involving techniques like enzyme‐linked immunosorbent assays or mass spectrometry (MS), and could not be performed within the short turnaround time in a breeding cycle, thus limiting the opportunities to enable selection for lower DON levels (Gaire et al., 2022). Recently, several simulated and empirical studies evaluated multi‐trait (MT) GP models that can leverage genetic correlations among different traits to improve PA for traits of interest (Gaire et al., 2022; Gill et al., 2021; Lado et al., 2018; J. Zhang et al., 2022a). Multiple studies have incorporated traits like days to heading, plant height (PH), and/or FDK as a covariate in MT models to predict DON (Gaire et al., 2021; Larkin et al., 2020; Moreno‐Amores et al., 2020; Schulthess et al., 2018; Steiner et al., 2017) and shown some improvement in the PA. Overall, these studies emphasized that FDK stands out to be the most important secondary trait to predict DON in MT models, and MT models consistently demonstrated superior performance compared to single‐trait (ST) GS. This creates novel avenues to evaluate MT GP models for DON using more accurate AI‐based FDK estimates as covariates.

In addition to high‐throughput robotics, various spectroscopic techniques have also been used recently to assist the phenotyping of complex traits in different crop species (Kalendar et al., 2022; Li et al., 2014). Unlike traditional spectroscopy techniques, hyperspectral imaging represents a novel nondestructive analytical approach that provides the ability to measure traits across a wide range of wavebands simultaneously, enhancing the analytical potential (Alvarez et al., 2021). Many studies have used hyperspectral imaging to assist phenotyping for a variety of traits in several crops, including FDK (Delwiche et al., 2011; Femenias et al., 2021; Shao et al., 2020). Further, these phenomics approaches have been integrated with ML and DL models to predict a variety of agronomic traits (Alzubaidi et al., 2021; Araus et al., 2018; Jackson et al., 2023; Robert et al., 2022). Nevertheless, there has been no report of utilizing hyperspectral imaging of FHB‐infected kernels coupled with GP to predict DON content. In the current study, we assessed the potential of hyperspectral imaging in improving the PA for DON by integrating it into the MT GP models. Further, several studies in wheat and other crops have suggested that phenomic prediction using image‐based spectral features can be equally effective or even surpass the performance of GS (Adak et al., 2023). Hence, it is intriguing to integrate hyperspectral imaging with ML/DL models to predict DON without relying on genomic information and avoiding tedious and expensive gas chromatography method.

The primary goal of this study was to integrate genomics and phenomics with ML methods to improve the prediction of FDK and DON in HWW to facilitate breeders in the selection of genotypes with low FDK and low DON. Based on this, the specific objectives of the study were (a) evaluating the PA of GP models using high‐throughput phenotyping‐derived FDK estimates, (b) assessing the performance of MT GS models including AI‐based FDK estimates as secondary traits to predict DON, (c) evaluating the incorporation of spectral information derived from hyperspectral imaging in MT GS models for DON prediction, and (d) evaluating direct use of the hyperspectral wavebands for phenomic prediction of DON via ML.

## 2 MATERIALS AND METHODS

### 2.1 Plant materials and phenotyping for FHB traits

To predict lines from earlier generations for FDK and DON, we used a diverse set of 250 breeding lines (F_4:9_ /F_4:8_ /F_4:7_ filial generation) from the winter wheat breeding program at South Dakota State University (SDSU). Four common FHB check cultivars including Emerson and Everest as the resistant controls and Flourish and Overley as the susceptible controls were used for FHB evaluation. Based on quality control (QC) of the genotype data, four lines were not used in downstream analysis.

The plant materials were planted in two independent experiments/FHB Field nurseries in 2022 at SDSU Agricultural Experimental Stations located in Brookings and Volga, SD. The planting and harvesting dates were September 30, 2021, and July 28–29, 2022, respectively. The set of 250 lines was planted in a randomized complete block design with two replications in each of the FHB nurseries. The experimental unit consisted of a 1 m single‐row plot with approximately 60 plants/row. Days to heading (HD) were recorded as Julian days when 50% of the plants in each row had completely emerged heads. PH was measured at maturity as the distance from the surface of the soil to the apex of the primary tiller excluding awns.

The disease nurseries were inoculated with *Fusarium graminearum*‐infested corn spawn (isolate SD‐FG1) as previously described in Halder et al. (2019). In brief, the infested corn spawn was uniformly spread at stage Feekes 10 and then at Feekes 10.1 to maximize the infection. In addition, to minimize disease escape, the lines were tagged at anthesis, and wheat heads were sprayed with a conidial suspension comprising 100,000 spores/mL at 50% anthesis. The field was mist irrigated using a sprinkler system every night (7:00 p.m. to 7:00 a.m.) for 2 min (every 10 minutes) to maintain high humidity and favor disease development. Phenotypic data were collected for disease incidence and severity 21 days postinoculation on at least 20 heads per row (each genotype) using the scale described by Stack and McMullen (2011). Disease incidence and severity were used to calculate disease index (DIS) as (incidence (INC) × severity (SEV)/100.

**FIGURE 1:**
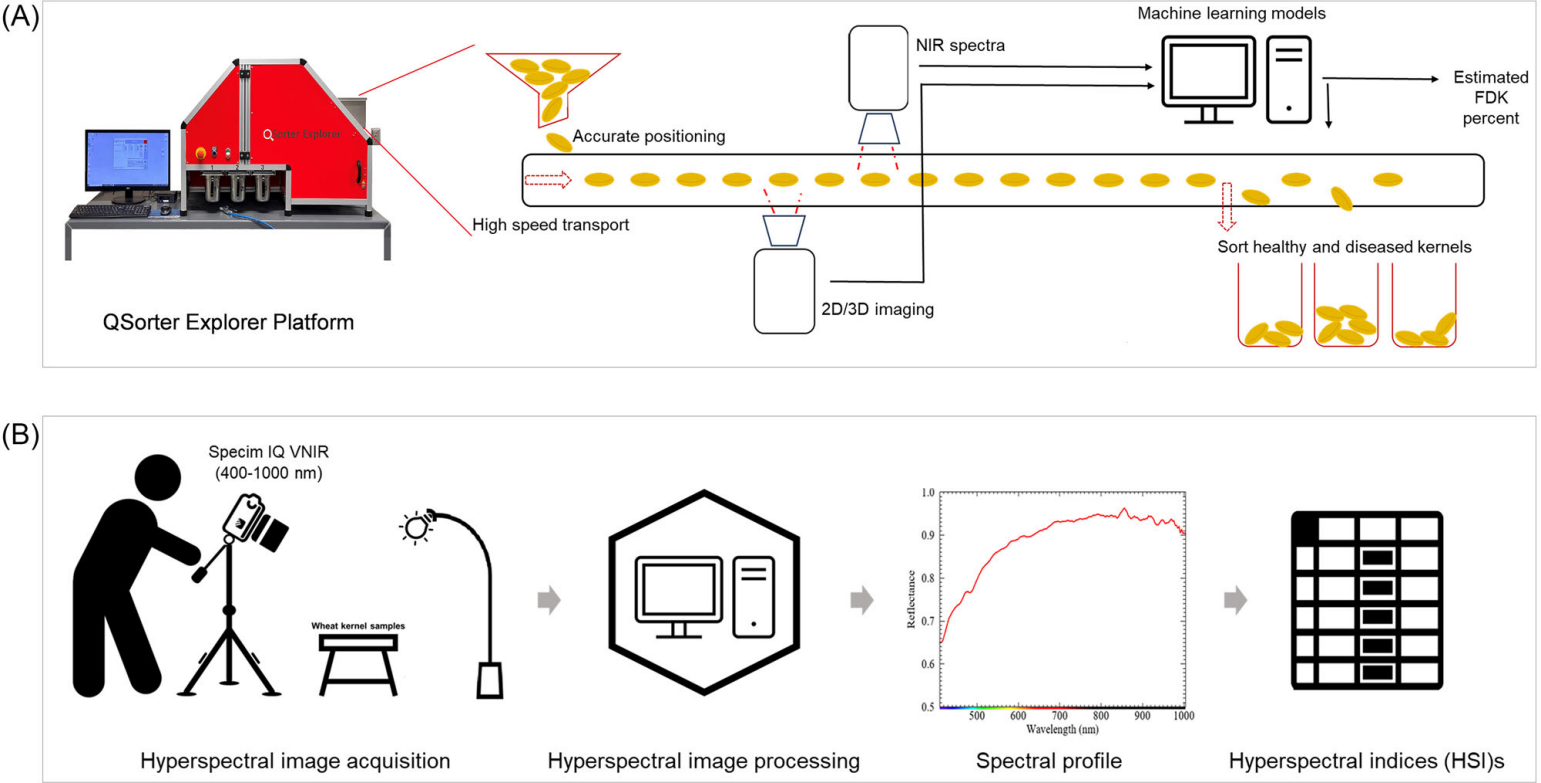
Visual representation of the phenotyping platform for (A) Fusarium‐damaged kernels (FDK) using QSorter Explorer and (B) deoxynivalenol (DON) using hyperspectral imaging.

Two postharvest FHB traits, namely FDK and DON, were recorded after harvesting the rows using a low airspeed thresher. FDK was measured using two different strategies. The first strategy involved trained personnel sampling the kernels for each line (in two technical replicates) and comparing them against a set of known FDK standards (Agronomic Crops Network, n.d.) to estimate the percentage FDK (referred to as FDK_V hereafter). The second strategy used an AI‐based automated seed sorter (QSorter Explorer) for measuring FDK based on two different algorithms. The QSorter Explorer is a sophisticated robot for single kernel analysis and sorting powered by cutting‐edge mechatronics and AI (Figure 1A). Briefly, the QSorter Explorer uses a 3D vision sensor and a high‐resolution near infrared (NIR) spectrometer to inspect each kernel and further uses AI‐based models to sort healthy and diseased kernels in real‐time. We employed QSorter in two ways to estimate FDK. First, we employed only a 3D imaging sensor to inspect each kernel and obtain the FDK value (FDK_QVIS). Second, both the 3D sensor and NIR spectrometer of the QSorter were used to inspect the appearance and spectral information for each kernel to estimate the FDK percentage (FDK_QNIR). In addition to FDK, the samples were also subjected to estimation of DON content (ppm) using the gas chromatography‐MS method (Simsek et al., 2013) at the Department of Plant Science, North Dakota State University.

### 2.2 Statistical analysis of the phenotype data

All the statistical analyses were performed in R statistical programming language (R Core Team, 2018) using different libraries. For the experimental design, best linear unbiased estimates (BLUEs) for various traits were estimated using the following model:

$$y_{ij}=\mu +E_{i}+G_{j}+e_{ij}$$

here $y_{ij}\;$ is the trait of interest, μ refers to overall mean, $E_{i}$ denotes the random effect of the *i* ^th^ experiment (referring to independent FHB nursery as two technical replicates within a nursery were averaged), $G_{j}\;$ is the fixed effect of the *j* ^th^ genotype, and $e_{ij}$ refers to the residual error effect of the *i* ^th^ experiment and *j* ^th^ genotype. The broad‐sense heritability (*H* ^2^) for FHB traits was estimated by fitting the genotypic effect from above equation as random using the following formula:

$$H^{2}=\frac{\sigma_{g}^{2}}{\sigma_{g}^{2}+\sigma_{e}^{2}/n\mathrm{Exp}}$$

where $\sigma_{g}^{2}$ and $\sigma_{e}^{2}$ are the genotype and error variance components, and *n*Exp refers to the number of experiments/nurseries. The above analysis was performed in R using the “MrBean” platform (Aparicio et al., 2023) based on “lme4” package (Bates et al., 2015). The correlations among traits were estimated and visualized based on the BLUEs for each trait using the psych library in R (William, 2024). All the model comparisons were visualized using “ggplot2” package in R (Wickham, 2016).

### 2.3 Genotyping‐by‐sequencing

The breeding lines were genotyped using genotyping‐by‐sequencing (GBS) as previously described (Gill et al., 2022). In brief, leaf tissues were used for DNA isolation using the hexadecyltrimethylammonium bromide method (Doyle & Doyle, 1987) and GBS libraries were prepared using the *PstI* and *MspI* restriction enzymes as described by Poland et al. (2012) and sequenced at the USDA Central Small Grain Genotyping Lab, Manhattan, KS. The sequencing data were used to call single‐nucleotide polymorphisms (SNPs) (Bradbury et al., 2007) using IWGSC Chinese Spring (CS) RefSeq v2.1 as the reference genome (Zhu et al., 2020). For QC, the SNPs with more than 30% missing values, minor allele frequency of <5%, and unmapped on a specific chromosome were removed, which yielded a total of 10,644 high‐quality SNPs. Those SNPs were imputed for missing datapoints using BEAGLE v4.1 (Browning & Browning, 2007) for further analysis. The principal component analysis (PCA) was performed for the genotypic data using “SNPRelate” package and visualized using “ggplot2” in R (Wickham, 2016).

### 2.4 GP models

#### 2.4.1 Single‐trait GP models

The ST genomic prediction for FDK and DON was performed using five different algorithms. The genomic BLUP (GBLUP) model (VanRaden, 2008) is the widely used GS model in plant breeding and used as a benchmark for comparison with other models. The GBLUP was implemented using a linear mixed model presented in the following equation:

y=1μ+Zu+e

where **y** is the vector (*n* × 1) of BLUE values for each trait; *μ* is the overall mean; **Z** is the incidence matrix for genotype effects; **u** is a random vector of genetic values with $u\;∼\;N(0,\;G\sigma_{g}^{2})$, where **G** is the genomic relationship matrix (VanRaden, 2008) and $\sigma_{g}^{2}$ is the additive genetic variance; and **e** is the vector of residual errors with $e\;∼\;N(0,\;\sigma_{e}^{2})$.

Apart from GBLUP, four commonly used Bayesian models, Bayes A (BA), Bayes B (BB), Bayes C (BC), and Bayesian ridge regression (BRR), were used for ST GP (Habier et al., 2011; Meuwissen et al., 2001; Pérez & De Los Campos, 2014). In contrast to GBLUP, the Bayesian models assume different prior distributions for estimating marker effects and variances overcomes the limitation of GBLUP, that is, homogenous shrinkage of marker effects (Lorenz et al., 2011; Pérez & De Los Campos, 2014), and these models have been widely evaluated for prediction of complex traits. A detailed account of the abovementioned Bayesian methods can be found in Montesinos‐López et al. (2022).

All the ST models were implemented in R package “BGLR” with 5000 burn‐ins and 15,000 iterations (https://github.com/gdlc/BGLR‐R/blob/master/inst/md/GBLUP.md)(Pérez & De Los Campos, 2014).

#### 2.4.2 Multi‐trait GP

An MT GP model was used with the trait of interest (FDK/DON) as primary trait and combination(s) of other traits as covariates. The model can be expressed as:

$$\;\left[\begin{array}{c} y_{1}\\ \vdots \\ y_{n} \end{array}\right]=\;\left[\begin{array}{c} I\\ \vdots \\ 0 \end{array}\;\begin{array}{c} 0\\ \vdots \\ I_{n} \end{array}\right]\;\left[\begin{array}{c} \mu_{1}\\ \vdots \\ \mu_{n} \end{array}\right]+\left[\begin{array}{c} Z\\ \vdots \\ 0 \end{array}\;\begin{array}{c} 0\\ \vdots \\ Z_{n} \end{array}\right]\;\left[\begin{array}{c} g_{1}\\ \vdots \\ g_{n} \end{array}\right]+\left[\begin{array}{c} e_{1}\\ \vdots \\ e_{n} \end{array}\right]$$

where **y** is the *n*‐dimensional vector of BLUEs for *n* traits, **I** and **Z** are the design matrices, $\mu_{t},$ *t *= 1 … *n*, refers to trait intercepts of *n* traits, $\left[\begin{array}{c} g_{1}\\ \vdots \\ g_{n} \end{array}\right]$ are the predicted genetic values assumed to be distributed as ∼MVN(0,∑⊗G) with **G** representing the genomic relationship matrix obtained following VanRaden (2008). The residuals of the MT model were assumed to be normally distributed as $\left[\begin{array}{c} e_{1}\\ \vdots \\ e_{n} \end{array}\right]$ ∼MVN(0,R⊗I). The matrices ∑ and **R** are the variance–covariance matrices for the genetic and residual effects between traits, with ∑ estimated as an unstructured variance–covariance matrix and **R** as a diagonal variance–covariance matrix. The MT model was implemented using the “MTM” package in an R environment (de los Campos & Grüneberg, 2016), employing the Gibbs sample algorithm with 15,000 iterations and 5000 burn‐ins.

#### 2.4.3 Combination of traits for MT model

We evaluated MT GP for three FDK estimates (FDK_V, FDK_QVIS, and FDK_NIR) by using them as primary traits and HD, PH, and/or DIS as secondary traits. Similarly, the MT model was used to evaluate PA for DON using a variety of combinations of secondary traits. For DON, we used HD, PH, and/or DIS as secondary traits. Thereafter, we expanded the combinations of secondary traits by including three types of FDK estimates (FDK_V, FDK_QVIS, and FDK_QNIR) along with HD, PH, and/or DIS. Finally, we evaluated a set of hyperspectral wavebands representing a wide range of wavelengths as secondary traits and DON as a primary trait in the MT model. Altogether, 21 different combinations of secondary traits were used in the MT model to predict DON. All the MT models were compared to the ST GBLUP model using Fisher's least significance difference test with Bonferroni correction using “agricolae” library in R (de Mendiburu, 2023).

#### 2.4.4 Cross‐validation for GP models

The PAs of the GP models were assessed by calculating the correlation between genome‐estimated breeding values and the observed phenotypic values of individuals in a testing set using a cross‐validation (CV) scheme. These CV schemes were followed based on real scenarios observed in plant breeding experiments. An 80:20 random CV approach, CV1, was used to evaluate the ST models, where 80% of the lines were used as the training set (had genotypic and phenotypic data) to train the model, and the remaining 20% were used as the testing set (only genotypic data) for prediction with 100 random repetitions. The PA of MT model was assessed using the CV2 scheme, where lines were split into an 80:20 ratio with 80% used as the training set, and the remaining 20% as the testing set (Gill et al., 2022). To train the model, we used genotypic data and the phenotypic data of the primary trait for the training set, along with phenotypic data of the secondary traits for training as well as the testing set with the objective of predicting the primary trait of the testing set. As fitting an MT model with several traits is quite extensive, we used 50 random repetitions of the CV2 approach to assess PA. Both the CV schemes have been illustrated and successfully employed in previous studies (Gaire et al., 2021).

### 2.5 Hyperspectral imaging and extraction of indices

A close‐range indoor hyperspectral imaging system, SPECIM IQ camera (SPECIM), was used to capture hyperspectral images of FHB‐infected wheat kernel samples (Figure 1B). It has a wavelength range of 397–1004 nm, a spatial resolution of 512 × 512 pixels, and a spectral resolution of 7 nm. It can capture hyperspectral imagery, radiometric calibration, data processing, and visualization. Hyperspectral imagery of the samples was collected indoors with halogen illumination. White reference panel (i.e., Lambertian surface) with SPECIM IQ imaging system was captured simultaneously with each sample during the image collection. This white reference data were used to transform the hyperspectral raw imagery digital numbers to reflectance values. This transformation was performed automatically based on built‐in functions. A total of 492 samples (246 genotypes in two replicates) were scanned using the SPECIM IQ camera, and each sample was imaged twice, making a total of 984 images.

The hyperspectral image cubes obtained from the SPECIM IQ camera were in the form of radiometrically corrected reflectance data. Due to the high noise levels, a few bands were removed from the reflectance images, leaving 196 spectral wavebands/indices. From each hyperspectral reflectance image, a region of interest (ROI) (i.e., 250 × 250 pixels), which only includes wheat kernel pixels, was extracted and used for further analysis. The average pixel values for each ROI chip image cube were further calculated and used as input variables for a one‐dimensional convolutional neural network (1D‐CNN) DL model that requires 1D input data.

### 2.6 Phenomic prediction model building and evaluation

The CNN algorithm is one of the most well‐known and classical approaches in DL (Alzubaidi et al., 2021; Zhou, 2020). It can often learn spatial, temporal, and spectral patterns from input data, demonstrating superior performance to fully connected feed‐forward deep neural network and conventional ML methods in many applications (Gu et al., 2018). Here, we investigate the potential of 1D‐CNN to predict DON solely based on hyperspectral wavebands. 1D‐CNN is often used for cases with 1D input variables; it can efficiently learn the sequential or spectral patterns and extract essential features from 1D input variables. In the current study, the input variables are 1D spectral profiles averaged from the wheat kernel hyperspectral chip images.

**FIGURE 2:**
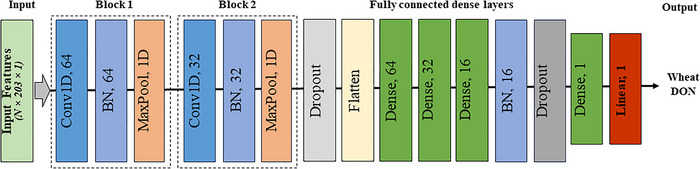
The input dimension, architecture, and output of the one‐dimensional convolutional neural network (1D‐CNN) deep learning model. *N* represents the number of samples used for model training or testing. BN represents batch normalization. DON, deoxynivalenol.

As shown in Figure 2, the optimal 1D‐CNN architecture was selected after investigating a variety of different architectures. Batch normalization of feature maps was employed. It often can effectively avoid gradient issues to potentially improve model performance (Ioffe & Szegedy, 2015). In addition, a dropout function was also incorporated to avoid overfitting issues. Rectified linear unit activation function was used in the convolutional and fully connected dense layers of DL architectures, and linear activation functions were adopted in the output layer. The samples were randomly split into training (70%) and testing (30%). The 30% unseen testing data were used to evaluate the model performance. Hyperparameter tuning was carried out during the training phase of 1D‐CNN model. Optimal learning rate, dropout rate, batch size, and so on, were selected to achieve the best model performance.

The performance of the model was evaluated using metrics such as root mean square error (RMSE) (Equation 1), relative root mean square error (RMSE%) (Equation 2), and the coefficient of determination (*R* ^2^) (Equation 3). Here, *yi* and *ŷi* refer to the measured and the predicted wheat DON content values, *ȳ* is the mean of measured wheat DON content values, and *n* is the total number of samples in the testing set.

(1) $$\mathrm{RMSE}=\sqrt{\frac{\sum_{i=1}^{n}{\left(\mathrm{yi}-\hat{y}i\right)}^{2}}{n-1}}$$

(2) $$\mathrm{RMSE}\%=\frac{\mathrm{RMSE}}{\bar{y}}\times 100$$

(3) $$R^{2}=\sqrt{1-\frac{\sum_{i=1}^{n}{\left(\mathrm{yi}-\hat{\mathrm{yi}}\right)}^{2}}{\sum_{i=1}^{n}{\left(\mathrm{yi}-\bar{y}\right)}^{2}}}\;$$

The epoch was set to 50 (50, 100, and 150 were tested), and the batch size was set to 32 (16, 24, and 32 were tested).

## 3 RESULTS

### 3.1 Phenotypic variation, correlation, and heritability for FHB traits

**FIGURE 3:**
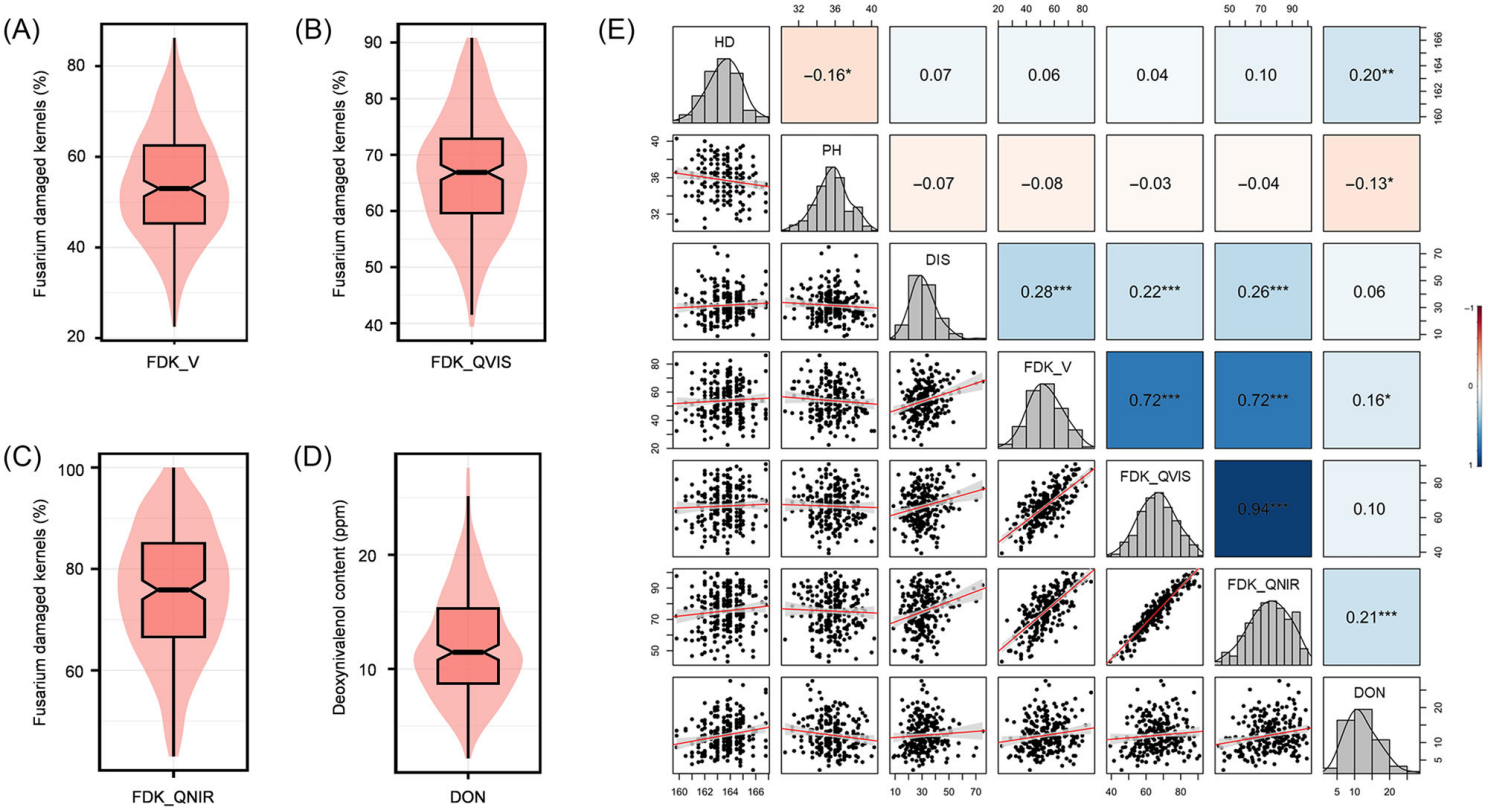
(A–D) Phenotypic distribution of different agronomic and Fusarium head blight traits using violin plots. (E) Pearson correlation coefficients among investigated traits using the best linear unbiased estimates (BLUEs) for various traits. The lower triangle shows the bivariate scatterplots with fitted lines and the upper triangle elucidates the correlation coefficients. Statistically significant differences are denoted by an asterisk (*) where *p ≤ 0.05, **p ≤ 0.01, and ***p ≤ 0.001. DIS, disease index; DON, deoxynivalenol; FDK_QNIR, vision‐ and spectroscopy‐based FDK from QSorter; FDK_QVIS, vision‐based FDK from QSorter; FDK_V, manually estimated FDK; HD, days to heading; PH, plant height.

A significant variation was observed among the tested genotypes for all three FHB traits, namely, DIS, FDK, and DON (Figure 3A–D; Table 1). In general, broad sense heritability (*H* ^2^) was moderate to high for various FHB traits and ranged from 0.68 for DON to 0.79 for DIS (Table 1). As described in the methods, we estimated FDK using three different methods. The *H* ^2^ for AI‐based FDK (FDK_QVIS and FDK_QNIR) was relatively higher than manually estimated FDK_V (Table 1). Moreover, we did observe a difference in the range of FDK among the three estimation methods. A higher mean FDK (FDK_QNIR; 75.40%) was observed using an AI‐based method compared to FDK_V (53.85%) (Table 1). A significant correlation was recorded between different sets of traits including among three FDK estimates as expected (Figure 3E). Overall, FDK showed a positive correlation with HD, DIS, and DON (Figure 3E). DON was positively correlated with HD and DIS, while negatively correlated with PH. Interestingly, we found a significantly higher positive correlation between DON and AI‐based FDK_QNIR (0.21) compared to FDK_V (0.16).

**TABLE 1:** Summary statistics and broad‐sense heritability (*H* ^2^) of various agronomic and Fusarium head blight traits. The estimates for genetic and residual variance were obtained from the mixed linear model for respective traits.

Trait	Mean	*H* ^2^	SD	Genetic variance (*σ* ^2^_g_)	Residual variance (*σ* ^2^_e_)
Days to heading (Julian days)	163.58	0.85	1.36	1.56	0.39
Plant height (cm)	90.57	0.73	4.92	2.84	1.51
Disease index (%)	31.63	0.79	10.22	80.12	40.30
FDK_V (%)	53.85	0.71	11.91	100.96	82.03
FDK_QVIS (%)	66.49	0.74	10.30	78.42	55.36
FDK_QNIR (%)	75.40	0.74	12.55	116.70	81.96
DON (ppm)	12.08	0.68	4.58	13.93	11.99

Abbreviations: DON, deoxynivalenol; FDK_QNIR, vision‐ and spectroscopy‐based FDK from QSorter; FDK_QVIS, vision‐based FDK from QSorter; FDK_V, manually estimated FDK; SD, standard deviation.

### 3.2 Genotyping analysis

GBS of 250 breeding lines yielded a total of 10,644 high‐quality SNP markers covering all 21 wheat chromosomes (Table S1). The highest number of SNPs were found in the B genome (4721), followed by the A genome (4124) and D genome (1799). PCA was performed using all the SNPs to investigate any stratification in the population and verify the relationship among lines from different stages of the breeding cycles (Figure S2). The first and second components explained 5.58% and 5.28% of the total variance, respectively. Strong population structure was not observed, suggesting close genetic relationships among the lines in the panel and the suitability of this panel for evaluating GP models.

### 3.3 Single‐trait GP for FDK and DON

**FIGURE 4:**
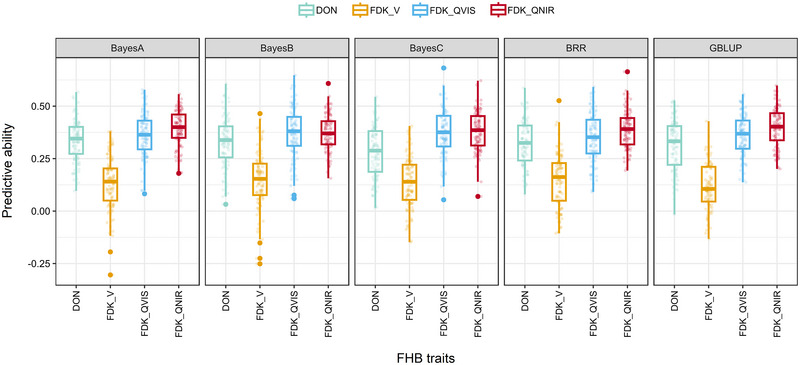
Boxplots comparing the predictive ability (PA) for four different Fusarium head blight (FHB) traits using five different single‐trait genomic prediction models. The four traits included three types of FDK estimated using different methods and deoxynivalenol (DON). BRR, Bayesian ridge regression; GBLUP, genomic BLUP.

The first objective of the study was to evaluate ST GP for different FHB traits and observe any differences in the performance of GP for visual (FDK_V) versus AI‐based (FDK_QVIS and FDK_QNIR) FDK estimations. We compared the PA for five different ST models to predict four different FHB traits, including FDK_V, FDK_QVIS, FDK_QNIR, and DON, using a CV approach, representing a real breeding scenario (Figure 4; Table S2). Overall, the results using all five ST models were comparable for all the traits with Bayesian models performing slightly better than the conventional GBLUP model in a few cases (Figure 4). For DON, the PA using five models ranged from 0.28 to 0.33 (Table S2), with the Bayes A model performing better than other models. Further, the PA for manually estimated FDK_V ranged from 0.11 to 0.15, with the Bayes B model having the highest PA. Interestingly, the AI‐based FDK traits, FDK_QVIS and FDK_QNIR, had significantly higher PA using all the models compared to manually estimated FDK_V (Figure 4). For instance, the PA for QVIS ranged from 0.35 using BRR to 0.37 using Bayes B. Similarly, for FDK_QNIR, we observed a slightly higher PA ranging from 0.37 to 0.40 using different ST models (Table S2).

### 3.4 Predictive abilities of MT GP models for FDK

**FIGURE 5:**
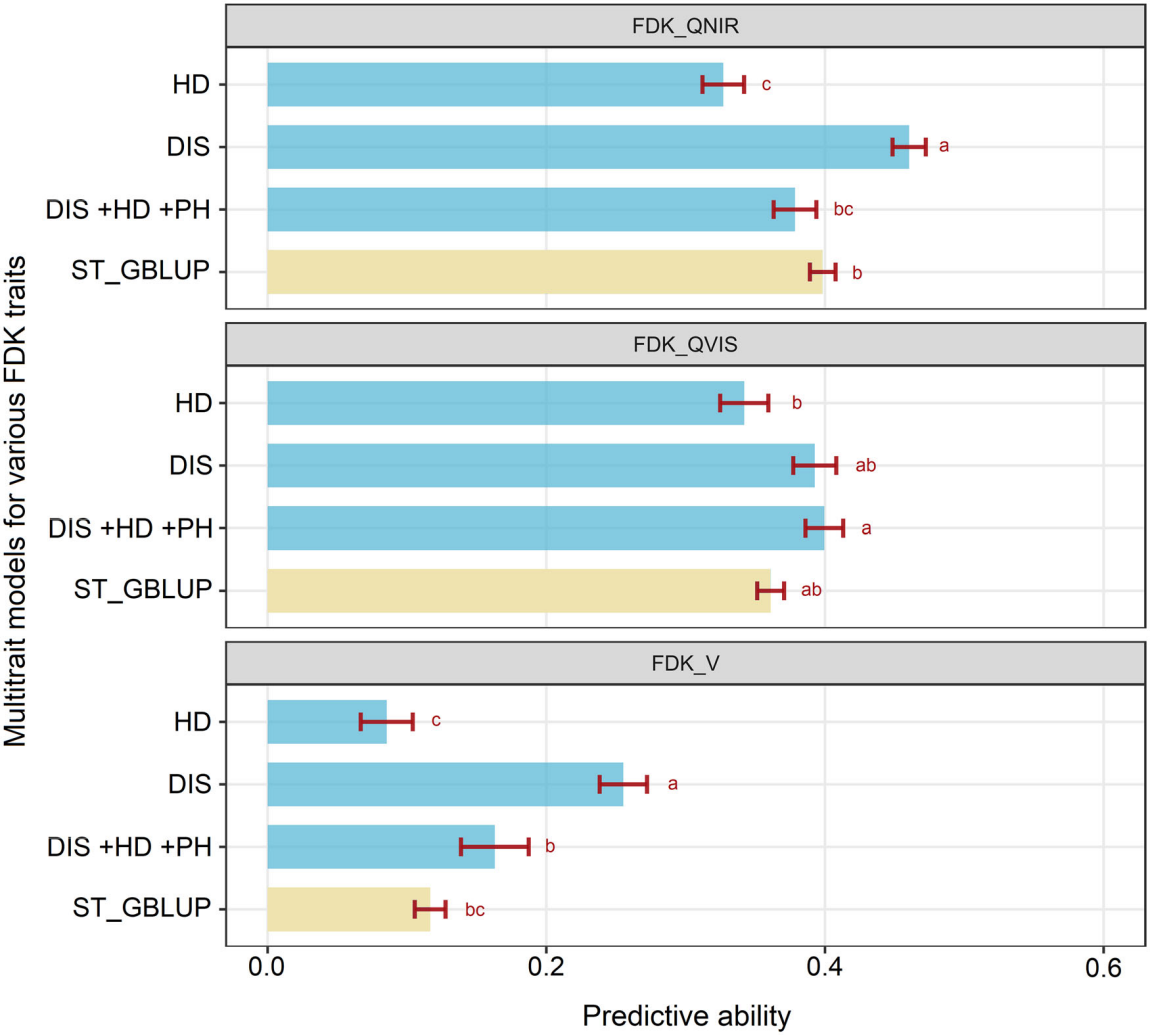
The predictive ability of three types of Fusarium‐damaged kernels (FDK) traits using a multi‐trait genomic prediction model with different combinations of secondary traits. The horizontal bars represent the mean predictive ability (PA), and the red error bars show the respective standard error. The baseline single‐trait model (ST‐GBLUP) has been represented using a yellow bar for comparison purposes. The different letters denote statistically different groups (*p* < 0.05). DIS, disease index; FDK_QNIR, vision‐ and spectroscopy‐based FDK from QSorter; FDK_QVIS, vision‐based FDK from QSorter; FDK_V, manually estimated FDK; HD, days to heading; PH, plant height.

We assessed the predictive abilities of MT GP for FDK_V, FDK_QVIS, and FDK_QNIR with PH, HD, and/or DIS as secondary traits in the MT model. For comparisons, ST‐GBLUP was used as a benchmark model. The PA using different combinations of covariates is presented in Figure 5 and Table S3. For FDK_V, we observed a significant increase in PA using DIS as a secondary trait in the MT model (0.25) as compared to ST‐GBLUP (0.11) (Figure 5). However, we did not see any improvement in PA for FDK_V while using HD and PH in the MT model. Further, the MT model showed only slight improvement in PA for FDK_QVIS when DIS was incorporated as a secondary trait. However, the inclusion of DIS into MT model to predict FDK_QNIR further increased the PA from 0.39 (ST GBLUP) to 0.46 (Figure 5). Nevertheless, we observed that the highest PA for FDK_V using MT GP models was 0.25, which was significantly lower even than the PA for FDK_QVIS/FDK_QNIR using the baseline ST‐GBLUP model, suggesting the usefulness of automated phenotyping for GP.

### 3.5 MT models for DON with different combinations of traits

**FIGURE 6:**
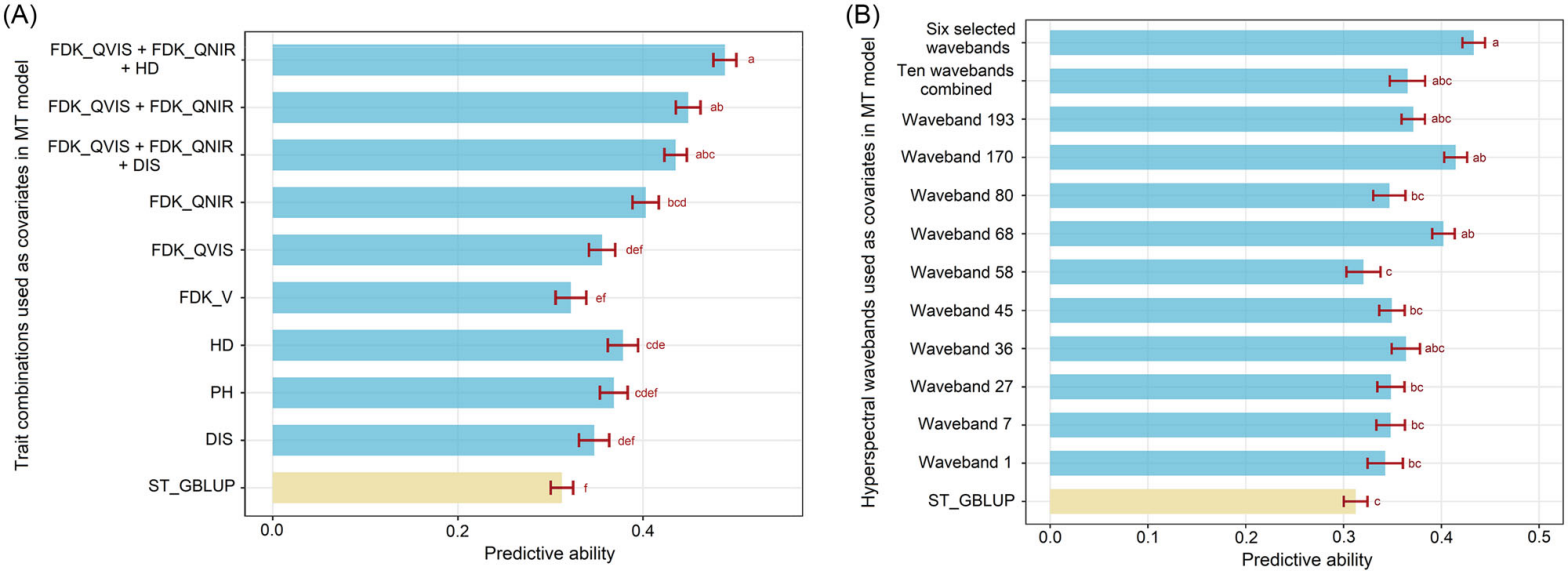
(A) Predictive abilities for deoxynivalenol (DON) using multi‐trait (MT) genomic prediction models with different combinations of traits. The *x*‐axis has the predictive accuracy (PA) of different models. The *y*‐axis elucidates the MT models to predict DON when different sets of combinations of secondary traits were included in the model. The horizontal bars represent the mean PA and the red error bars show the respective standard error. The baseline single‐trait model (ST‐GBLUP) has been represented using yellow bar for comparison purposes. The different letters denote statistically different groups (*p* < 0.05). (B) Comparison of multi‐trait genomic prediction models for DON that used various combinations of hyperspectral image bands as covariates. The *x*‐axis has the PA of different models. The *y*‐axis elucidates the MT models to predict DON when different combinations of hyperspectral wavebands as secondary traits were included in model. DIS, disease index; FDK_QNIR, vision‐ and spectroscopy‐based FDK from QSorter; FDK_QVIS, vision‐based FDK from QSorter; FDK_V, manually estimated FDK; HD, days to heading; PH, plant height.

The MT model for DON included a variety of combinations of traits as covariates, including agronomic traits, DIS, and different types of FDK traits. Here, we were intrigued to see if the inclusion of AI‐based FDK in the MT model is better than manually estimated FDK for the prediction of DON. The ST‐GBLUP for DON (PA = 0.32) was used as a baseline to compare any improvement using the MT models. In general, we saw a significant increase in PA using the MT model compared to the baseline ST model (Figure 6A; Table S4). Among various agronomic traits as covariates in the MT model, inclusion of HD increased the PA for DON to 0.39. We did not see any significant improvement in PA for DON while using DIS or manual FDK (FDK_V) as secondary traits (Figure 6A). Interestingly, we observed a significant leap in PA when AI‐based FDK traits were introduced in the MT model for DON. Though the inclusion of one secondary trait like FDK_QNIR in the MT model yielded a PA of 0.40 for DON; however, inclusion of both FDK_QVIS and FDK_QNIR raised the PA to 0.45, suggesting around 45% improvement over the baseline ST model. Moreover, the PA reached up to 0.49 when AI‐based FDK traits were combined with HD, resulting in around 50% improvement over the ST‐GBLUP (Figure 6A).

### 3.6 Hyperspectral image bands assisted MT models in predicting DON

We studied the usefulness of hyperspectral wavebands as a substitute for agronomic or FDK traits in the MT model. As described in the methods, 196 wavebands were extracted from hyperspectral images of the sampled kernels that were later used for the prediction of DON. We estimated the Pearson correlation between 196 bands and the DON value of each sample and observed a moderate correlation for several bands representing different wavelengths (Figure S3).

From 196 wavebands, we chose 10 different bands (Bands 1, 7, 27, 36, 45, 58, 68, 80, 170, and 193) representing a variety of wavelength ranges, based on correlation with DON and the collinearity among the hyperspectral wavebands. The selected 10 bands were then evaluated as covariates in the MT GP in different combinations (Figure 6B). Initially, each of the 10 wavebands was included individually as secondary traits in the bivariate MT model. Subsequently, only six of the 10 wavebands (Bands 1, 7, 58, 80, 170, and 193), and, finally, all 10 bands were used as covariates in the MT model. While the 10 bands were tested one by one in the MT model, two bands showed a significant increase in PA for DON, with Band 170 yielding a PA of 0.41 and Band 68 resulting in a PA of 0.40 (Figure 6B). Further, for a comprehensive evaluation, all 10 bands were collectively employed in the full MT model to assess PA, but we did not observe substantial improvement (0.37) (Figure 6B; Table S5). Interestingly, when only a subset of six bands (selected based on correlation) was included in the model, the PA reached 0.43 compared to 0.31 in case of the ST‐GBLUP model (Figure 6B).

### 3.7 Phenomic prediction for DON based on hyperspectral imaging

**FIGURE 7:**
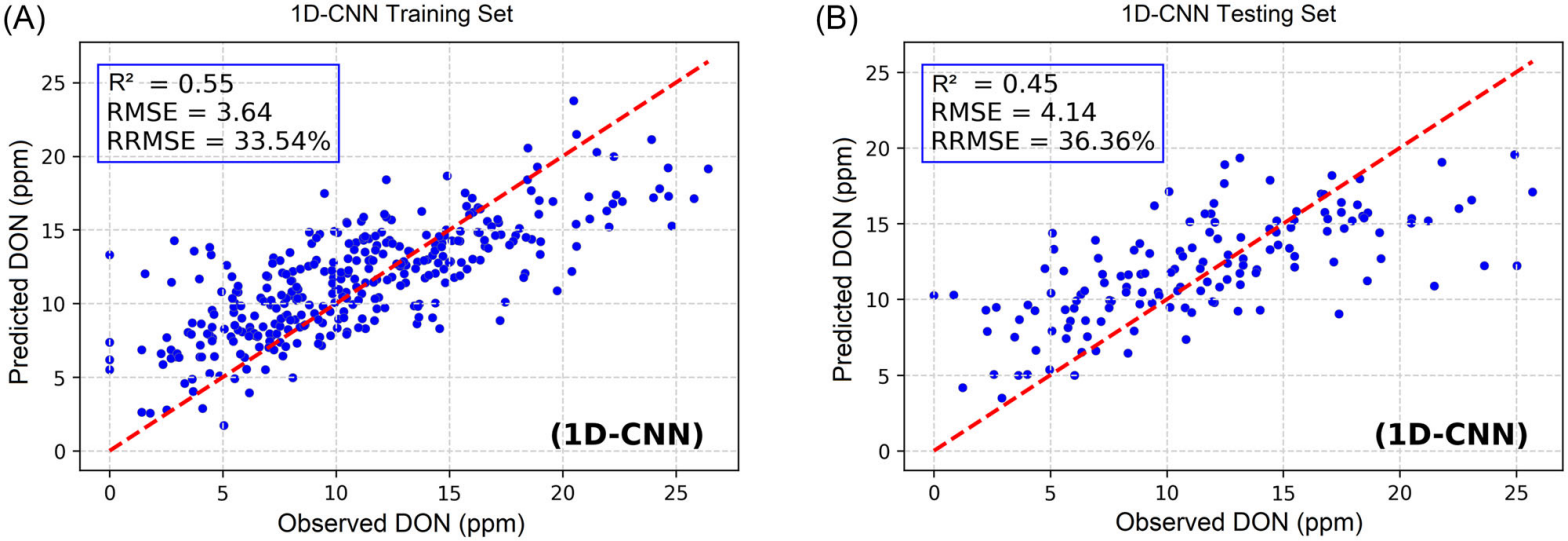
Phenomic prediction of deoxynivalenol (DON) based one‐dimensional convolutional neural network (1D‐CNN) model employing 196 hyperspectral wavebands from 984 hyperspectral images. The whole set of 984 images was split into two sets (training and testing) in a 70:30 ratio. The 1D‐CNN was first trained on the training set and then independently validated on the testing set. The two scatterplots present the model statistics for the training set (A) and the independent testing set (B). RMSE, root mean square error; RRMSE, relative root mean square error.

Finally, we used the hyperspectral wavebands obtained from 984 images of flour samples in the phenomic prediction of DON using a DL prediction model. Here, no genomic information was incorporated in the model and DON was predicted solely based on 196 wavebands using a 1D‐CNN model. The prediction performance of the model was determined using the coefficient of determination (*R* ^2^) and RMSE (Figure 7). In the training set, using the 1D‐CNN model, we observed an *R* ^2^ of 0.55 with an RMSE of 3.64 (Figure 7A). Further, the model was validated on an independent validation set, where the model yielded *R* ^2^ of 0.45 with RMSE of 4.4 (Figure 7B), thus performing at par with most of the MT GP models used in this study.

## 4 DISCUSSION

Resistance to FHB is essential in varietal development in the US HWW breeding programs. However, the accurate selection of traits contributing to FHB resistance in a breeding program has been hindered by expensive and time‐consuming phenotyping requirements. Furthermore, the short turnaround time of the winter wheat breeding programs in the northern Great Plains region makes it impractical to select for the postharvest traits, including FDK and DON. In this study, we envisaged the integration of GS, phenomics, and ML to improve the predictive abilities for FDK and DON to assist the breeders in the improvement of FHB resistance.

FDK, also referred to as visual scabby kernels, is estimated in most breeding programs by visually comparing the kernels to a set of standard images or standard samples, making it highly subjective and prone to human bias (Ackerman et al., 2022). For instance, we selected a set of 100 samples with varying FDK levels and got it rated by two different trained personnel. Interestingly, we observed a correlation of 0.77 between the ratings of two evaluators, clearly suggesting the possibility of human bias.

In the current study, we utilized a novel AI‐assisted single‐kernel analyzer (QSorter Explorer) that uses kernel morphology (based on visual appearance) extracted from the three‐dimensional images of each kernel along with NIR spectra of the kernel. We used QSorter to evaluate FDK in two different ways. At first, the analyzer used the single‐kernel images to classify the kernels into diseased and healthy classes based on visual defects and then calculated the FDK percentage based on two classes (FDK_QVIS). In the second approach, the QSorter used visual appearance along with NIR spectral information from each kernel to classify them into two classes and obtain FDK (FDK_QNIR). Our results show that the AI‐based FDK estimates (FDK_QVIS and FDK_QNIR) had slightly higher heritability (0.74) compared to manually estimated FDK (FDK_V, 0.71) (Table 1), as previously reported by Wu et al. (2023) based on analysis of FDK on bulk seed samples. Similarly, we observed higher mean FDK using AI‐based methods as reported by Ackerman et al. (2022). Further, our results demonstrate that the AI‐based FDK shows a higher correlation (0.21) with DON compared to the manually estimated FDK (0.16) (Figure 3E), as shown in previous reports (Ackerman et al., 2022). Overall, our study demonstrates that applications of AI‐ and vision‐based tools are helpful in improving the estimation of FDK and overcoming the drawbacks associated with the traditional approach, as suggested by similar studies for other traits in various crops (Meraj et al., 2024; Mochida et al., 2019; Mutka & Bart, 2015). Further, precise estimation of FDK provides room for an opportunity to better exploit GP for this trait.

Next, we compared the PA of ST GP models to predict FDK_V, FDK_QVIS, and FDK_QNIR. The results from five GP methods consistently showed that AI‐based FDK_QNIR had the highest PA, with an improvement of about 100% over manual evaluation FDK_V (Table S2). This improvement in PA could be due to unbiased and more accurate phenotyping and thus improved heritability when AI‐based phenotyping is involved. As the PA for FDK_V was lower compared to the PA for FDK_QVIS and FDK_QNIR in ST GP, we also evaluated an MT GP model to predict FDK_V using HD and/or DIS as covariates. Interestingly, even the MT GP models for FDK_V could not surpass the PA observed for a baseline ST GP model for AI‐based FDK traits (Figure 5). These results suggest the usefulness of exploiting vision‐based and automated phenotyping for FDK and its suitability in GP.

In the second GS approach, we evaluated MT GP models to assess the PA for DON by incorporating manually estimated FDK (FDK_V), or FDK derived from an AI‐based platform (FDK_QVIS and FDK_QNIR) along with agronomic traits as covariates to predict DON. Our results demonstrated around 50% improvement in PA for DON using MT models compared to the baseline ST GBLUP model (Figure 6A; Table S4), which is in agreement with several previous studies on exploiting MT GP models for FHB traits (Gaire et al., 2022; Larkin et al., 2020; Wu et al., 2023; J. Zhang et al., 2022a). The highest PA was achieved using the MT model having AI‐based FDK traits as covariates. Unlike our findings, Wu et al. (2023) did not observe any improvement in MT prediction of DON using AI‐based FDK and attributed it to the misclassification of kernels by the neural network. In our case, the AI‐based platform used 3D imaging and NIR spectral information from single kernels, which resulted in better classification of diseased and healthy kernels, an accurate estimate of FDK, and eventually a high correlation with the DON.

Hyperspectral imaging combined with different ML/DL models has proven to be a promising technology for automated nondestructive phenotyping of various traits (Cheshkova, 2022; Li et al., 2014). In our study, we integrated hyperspectral imaging of kernels harvested from FHB nursery with MT GP models, as well as used it independently in the phenomic prediction by directly predicting DON using DL models. To the best of our knowledge, this is the first study to evaluate the potential of close‐range hyperspectral imaging in MT GS for DON in wheat.

In the first scenario, we used MT GP to predict DON when hyperspectral wavebands were used as covariates and no other agronomic or FHB‐related traits (as mentioned in the Methods section) were included in the MT GP model. As it is tedious to use all wavebands in building an MT GP model, we extracted only 10 wavebands from a total of 196 wavebands based on correlation and representing the complete wavelength range. We observed a significant increase in PA for DON when MT GP models included wavebands as covariates; however, the results suggested that only few wavebands from certain wavelengths are useful in MT GP models rather than including all the wavebands (Table S5). Different studies have recommended different hyperspectral ranges for detection of fungal infection or Fusarium in wheat. For instance, Berman et al. (2007) recommend a 420–1000 nm region, whereas Singh et al. (2007) recommend a 1000–2500 nm region. However, Cheshkova (2022) reviewed a set of spectral wavelength ranges for plant disease detection and suggested that specific ranges/spectra can be more useful for the detection of a variety of plant diseases in different crop species. In this regard, our results also indicate the potential of including two selected wavebands in MT GP models as covariates, which yielded a comparable PA for DON compared to MT models built with various agronomic or FHB traits. Nevertheless, further work is needed to evaluate a wider hyperspectral range to provide better insights about the most effective wavelength or range for DON estimation and that would facilitate more efficient prediction of DON when utilized in MT GP models.

In the second scenario, complete 196 wavebands from 984 images were directly used for phenomic prediction of DON by leveraging DL based 1D‐CNN model. We achieved a comparable performance for predicting DON using 1D‐CNN in model training (*R* ^2^ = 0.55) and independent validation (*R* ^2^ = 0.45) (Figure 7) to that of different MT GP models evaluated in the current study (Figure 7). Moreover, the phenomic prediction (*R* ^2^ = 0.45) outperformed all the ST GP models for DON evaluated in this study (Figure 7; Table S2), which is in corroboration with recent reports from various crop species where phenomic prediction performed better or at par with GP (Adak et al., 2023; Jackson et al., 2023; Robert et al., 2022; Winn et al., 2023). These findings suggest a promising avenue for estimating DON based on hyperspectral imaging, where breeders can quickly inform their selection based on predicted DON. Though no similar study has been reported in wheat, Su et al. (2021) investigated the viability of employing hyperspectral imaging (382–1030 nm) to develop a fast and nondestructive methodology for testing DON in barley kernels. They used full wavelength with locally weighted partial least squares regression to attain an *R* ^2^ of 0.728 and an RMSE of 3.802, suggesting room for improvement in wheat. Also, further research based on different materials and improved modeling is needed to lower the error in DON prediction and exploit this strategy on a routine basis. In general, our results suggest a potential application of hyperspectral imaging‐based phenomic prediction for DON and necessitate further evaluation for fine‐tuning the models by utilizing larger datasets and more robust DL approaches.

## 5 CONCLUSION

FHB poses a severe threat to global wheat production and food safety. However, phenotyping FHB traits such as FDK and DON is laborious, time‐consuming, and expensive, which hinders breeding efforts to develop FHB‐resistant cultivars. This study shows that AI‐assisted phenotyping for FDK could improve the PA for FDK itself, and for DON when used as a covariate in MT GP models, demonstrating its potential application in wheat breeding. Further, we observed hyperspectral imaging in conjugation with ML and DL models as a novel avenue to estimate DON in FHB‐infected wheat kernels with prediction accuracy comparable to GP models, suggesting a great potential for hyperspectral imaging‐assisted phenomic prediction in improving selection accuracy for these traits in wheat breeding.

## AUTHOR CONTRIBUTIONS

**Subash Thapa**: Conceptualization; data curation; formal analysis; investigation; software; validation; visualization; writing—original draft. **Harsimardeep S. Gill**: Data curation; formal analysis; investigation; methodology; software; visualization; writing—original draft. **Jyotirmoy Halder**: Formal analysis; investigation; methodology; writing—review and editing. **Anshul Rana**: Investigation; writing—review and editing. **Shaukat Ali**: Investigation; writing—review and editing. **Maitiniyazi Maimaitijiang**: Formal analysis; writing—review and editing. **Upinder Gill**: Formal analysis; writing—review and editing. **Amy Bernardo**: Investigation; writing—review and editing. **Paul St. Amand**: Formal analysis; software; writing—review and editing. **Guihua Bai**: Investigation; writing—review and editing. **Sunish K. Sehgal**: Conceptualization; data curation; funding acquisition; methodology; project administration; resources; supervision; validation; writing—original draft.

## CONFLICT OF INTEREST STATEMENT

The authors declare no conflicts of interest.

## Supporting information


**Supplementary Table S1**. The distribution of 10,644 SNPs across 21 wheat chromosomes in the set of 246 breeding lines.
**Supplementary Table S2**. The mean prediction ability along with standard deviation (SD) and standard error (SE) for five FHB traits using different single‐trait genomic prediction models. DIS, disease index; DON, deoxynivalenol; FDK_V, Fusarium damaged kernel manual; FDK_QVIS, Fusarium damaged kernel QSorter Visual; FDK_QNIR, Fusarium damaged kernel QSorter Near‐infrared.
**Supplementary Table S3**. The mean prediction ability along with standard deviation (SD) and standard error (SE) for FDK using MT GP models. FDK_V, Fusarium damaged kernel manual; FDK_QVIS, Fusarium damaged kernel QSorter Visual; FDK_QNIR, Fusarium damaged kernel QSorter Near‐infrared.
**Supplementary Table S4**. The mean prediction ability along with standard deviation (SD) and standard error (SE) for DON using MT GP models. DIS, disease index; PH, plant height; HD, days to heading; FDK_V, *Fusarium* damaged kernel manual; FDK_QVIS, *Fusarium* damaged kernel QSorter Visual; FDK_QNIR, *Fusarium* damaged kernel QSorter Near‐infrared.
**Supplementary Table S5**. The mean prediction ability along with standard deviation (SD) and standard error (SE) for DON integrating hyperspectral image bands (HIB)s with MT GP model.
**Supplementary Figure S1**. Schematic representation of the timeline for analysis of various FHB traits in the winter wheat breeding pipeline in the northern Great Plains. Red cross indicates the non‐feasibility of the evaluation due to the short turn around in the breeding program.
**Supplementary Figure S2**. Biplot of the first two components of the principal component analysis (PCA) of 246 genotypes based on 10,644 SNPs. The first two PCs explained 5.58% and 5.28% of the total variation, respectively.
**Supplementary Figure S3**. Correlation (normalized) between the hyperspectral bands representing different wavelengths and DON value.

## Data Availability

The datasets generated in the study are presented in the manuscript or the supporting information associated with the manuscript.
