# Supplementary material for: Integrating genomics, phenomics, and deep learning improves the predictive ability for Fusarium head blight–related traits in winter wheat
Source: Plant Genome. 2024 Jun 9;17(3):e20470. doi: 10.1002/tpg2.20470 (PMC12807346; doi:10.1002/tpg2.20470)

Supplementary Information

Integrating genomics, phenomics, and deep learning improves the predictive ability for Fusarium head blight-related traits in winter wheat

Subash Thapa[1], Harsimardeep Gill[1], Jyotirmoy Halder[1], Anshul Rana[1], Shaukat Ali[1], Maitiniyazi Maimaitijiang[2], Upinder Gill[3], Amy Bernardo[4], Paul St. Amand[4], Guihua Bai[4], and Sunish K Sehgal[1*]

Supplementary Tables

Supplementary Table S1. The distribution of 10,644 SNPs across 21 wheat chromosomes in the set of 246 breeding lines.

Sub-genome	Chromosome	Number of SNPs	%SNPs
A	1	552	
	2	563	
	3	623	
	4	421	
	5	580	
	6	520	
	7	865	
Subtotal A		4124	38.8
B	1	609	
	2	785	
	3	866	
	4	301	
	5	713	
	6	780	
	7	667	
Subtotal B		4721	44.3
D	1	275	
	2	469	
	3	347	
	4	63	
	5	168	
	6	232	
	7	245	
Subtotal D		1799	16.9
Grand Total		10644	

Supplementary Table S2. The mean prediction ability along with standard deviation (SD) and standard error (SE) for 5 FHB traits using different single-trait genomic prediction models. DIS, disease index; DON, deoxynivalenol; FDK_V, *Fusarium* damaged kernel manual; FDK_QVIS, *Fusarium* damaged kernel QSorter Visual; FDK_QNIR, *Fusarium* damaged kernel QSorter Near infrared.

**Trait**	**Model**	**PA**	**SD**	**SE**
DIS	BayesA	0.065	0.124	0.012
	BayesB	0.071	0.123	0.012
	BayesC	0.069	0.144	0.014
	BRR	0.082	0.127	0.013
	GBLUP	0.090	0.122	0.012
DON	BayesA	0.336	0.098	0.010
	BayesB	0.331	0.120	0.012
	BayesC	0.282	0.123	0.012
	BRR	0.323	0.109	0.011
	GBLUP	0.312	0.121	0.012
FDK_V	BayesA	0.133	0.125	0.013
	BayesB	0.149	0.134	0.013
	BayesC	0.138	0.122	0.012
	BRR	0.146	0.128	0.013
	GBLUP	0.117	0.110	0.011
FDK_QVIS	BayesA	0.361	0.103	0.010
	BayesB	0.370	0.121	0.012
	BayesC	0.370	0.111	0.011
	BRR	0.349	0.115	0.012
	GBLUP	0.361	0.096	0.010
FDK_QNIR	BayesA	0.395	0.083	0.008
	BayesB	0.371	0.088	0.009
	BayesC	0.380	0.092	0.009
	BRR	0.390	0.089	0.009
	GBLUP	0.398	0.092	0.009

Supplementary Table S3. The mean prediction ability along with standard deviation (SD) and standard error (SE) for FDK using MT GP models. FDK_V, *Fusarium* damaged kernel manual; FDK_QVIS, *Fusarium* damaged kernel QSorter Visual; FDK_QNIR, *Fusarium* damaged kernel QSorter Near infrared.

Primary trait	Covariate(s)	PA	SD	SE
FDK_V	DIS + HD + PH	0.16	0.172	0.024
	DIS	0.25	0.120	0.017
	HD	0.08	0.132	0.019
FDK_QVIS	DIS + HD + PH	0.40	0.096	0.014
	DIS	0.39	0.109	0.015
	HD	0.34	0.122	0.017
FDK_QNIR	DIS + HD + PH	0.38	0.108	0.015
	DIS	0.46	0.084	0.012
	HD	0.33	0.106	0.015

Supplementary Table S4. The mean prediction ability along with standard deviation (SD) and standard error (SE) for DON using MT GP models. DIS, disease index; PH, plant height; HD, heading days; FDK_V, *Fusarium* damaged kernel manual; FDK_QVIS, *Fusarium* damaged kernel QSorter Visual; FDK_QNIR, *Fusarium* damaged kernel QSorter Near infrared.

**Primary trait**	**Covariate(s)**	**PA**	**SD**	**SE**
DON	ST_GBLUP	0.31	0.121	0.012
DON	DIS	0.35	0.116	0.016
DON	PH	0.37	0.106	0.015
DON	HD	0.38	0.115	0.016
DON	FDK_V	0.32	0.118	0.017
DON	FDK_QVIS	0.36	0.099	0.014
DON	FDK_QNIR	0.40	0.101	0.014
DON	FDK_QVIS + FDK_QNIR + DIS	0.43	0.086	0.012
DON	FDK_QVIS + FDK_QNIR	0.45	0.094	0.013
DON	FDK_QVIS + FDK_QNIR + HD	0.49	0.088	0.012

Supplementary Table S5. The mean prediction ability along with standard deviation (SD) and standard error (SE) for DON integrating hyperspectral image bands (HIB)s with MT GP model.

Primary trait	Covariate(s)	PA	SD	SE	Group
DON	Selected	0.43	0.08	0.01	a
DON	Combined_10	0.37	0.13	0.02	abc
DON	Band 193	0.37	0.09	0.01	abc
DON	Band 170	0.41	0.08	0.01	ab
DON	Band 80	0.35	0.11	0.01	bc
DON	Band 68	0.40	0.08	0.01	ab
DON	Band 58	0.32	0.12	0.01	c
DON	Band 45	0.35	0.09	0.01	bc
DON	Band 36	0.36	0.10	0.01	abc
DON	Band 27	0.35	0.1	0.01	bc
DON	Band 7	0.35	0.1	0.01	bc
DON	Band 1	0.34	0.13	0.01	bc
DON	ST_GBLUP	0.31	0.12	0.01	c

Supplementary Figures

Supplementary Figure S1. Schematic representation of the timeline for analysis of various FHB traits in the winter wheat breeding pipeline in the northern Great Plains. Red cross indicates the non-feasibility of the evaluation due to short turn around in the breeding program.

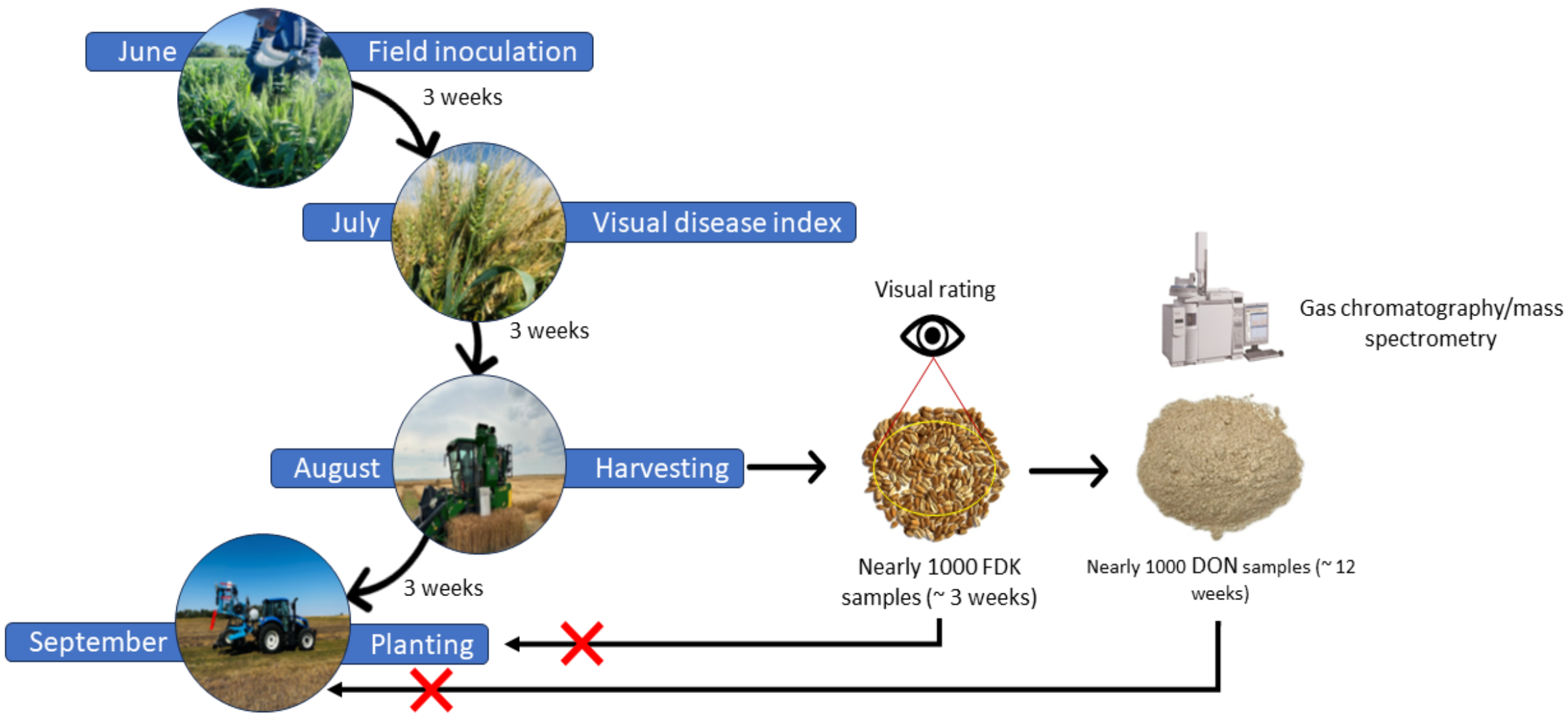

Supplementary Figure S2. Biplot of the first two components of the principal component analysis (PCA) of 246 genotypes based on 10,644 SNPs. The first two PCs explained 5.58% and 5.28% of the total variation, respectively.

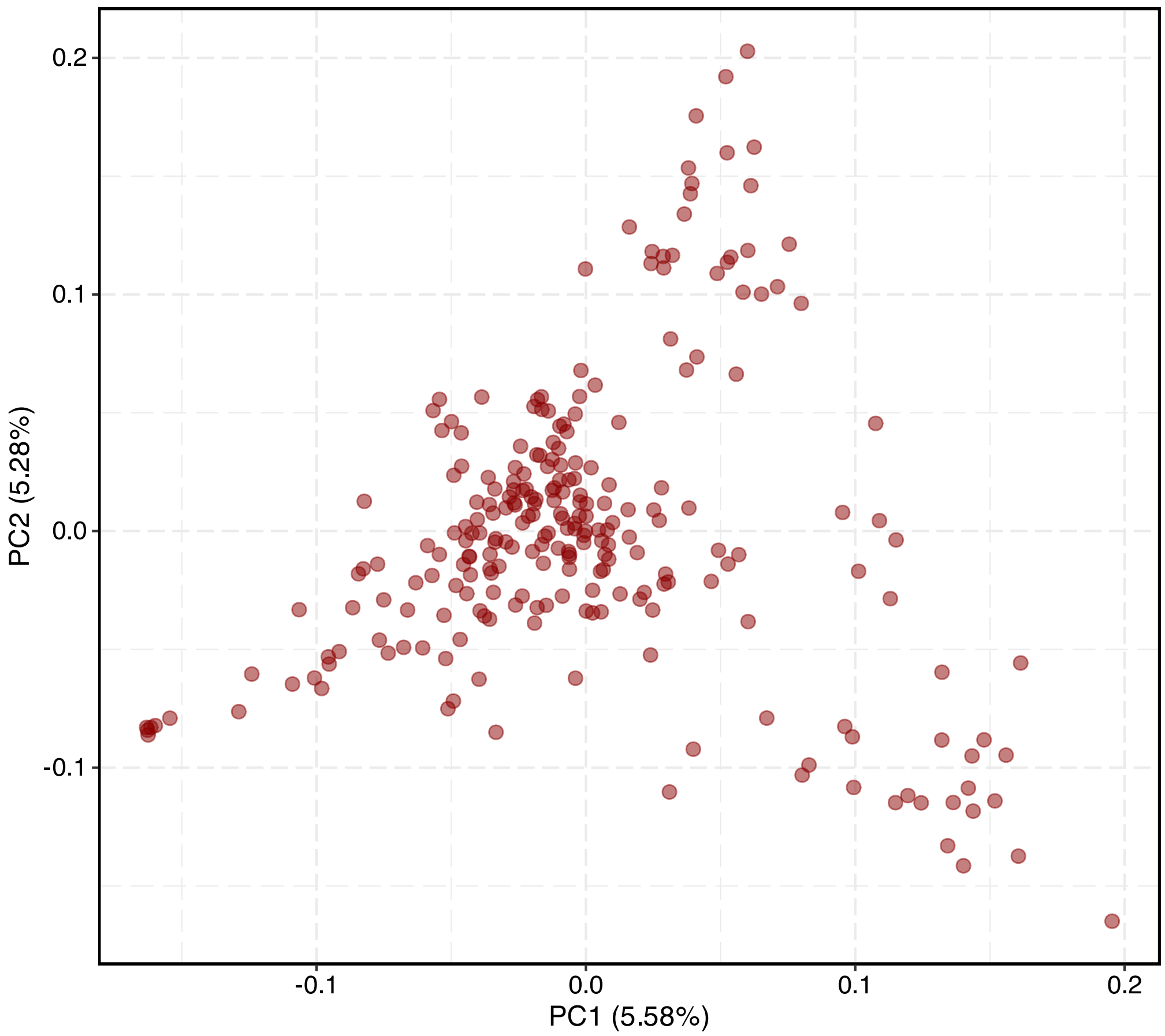

Supplementary Figure S3. Correlation (normalized) between the hyperspectral bands representing different wavelengths and DON value.

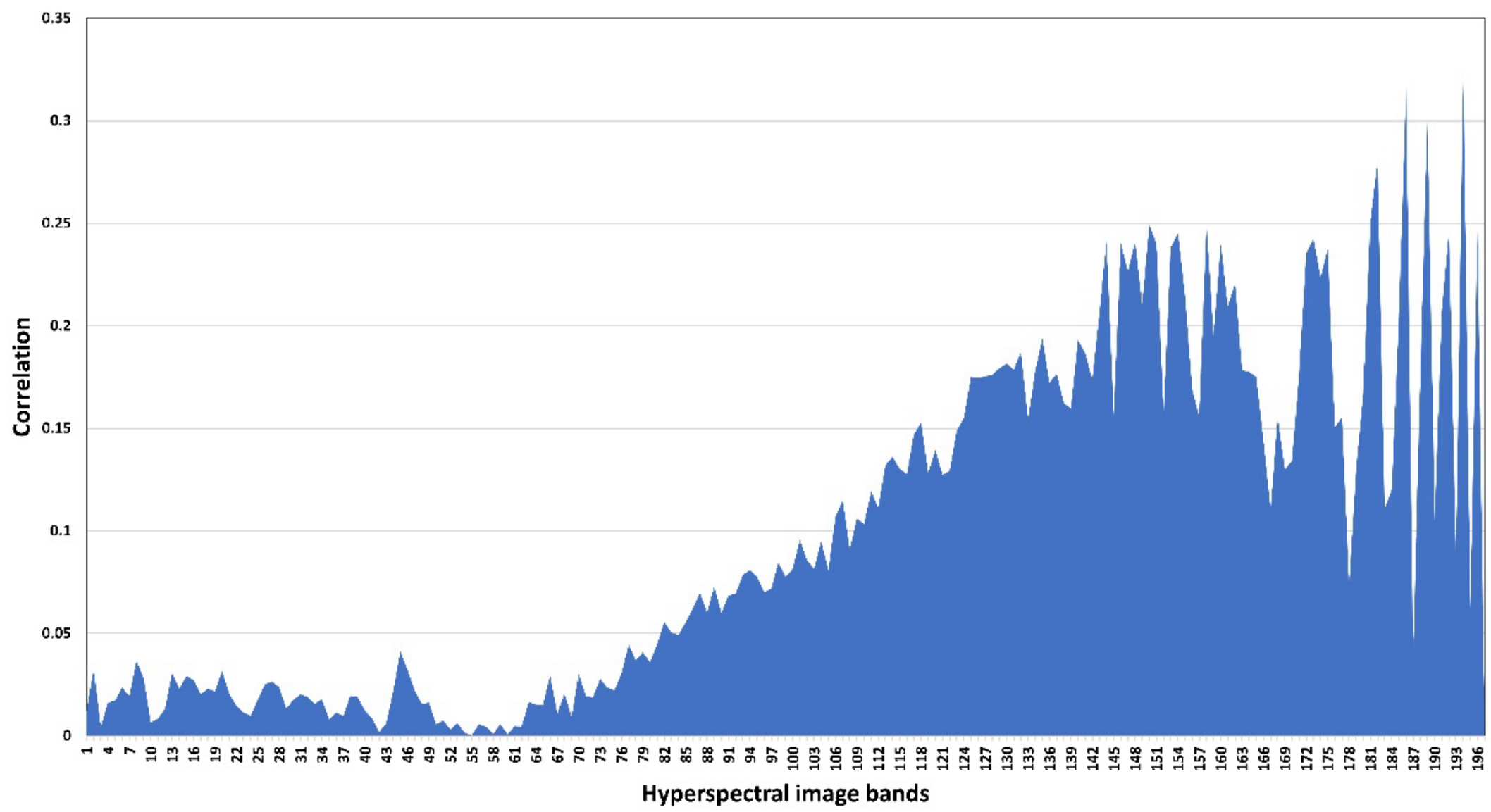

Supplement: Supplementary file 1 [file TPG2-17-e20470-s001.pdf]
